# Macrosomia, Perinatal and Infant Mortality in Cree Communities in Quebec, 1996-2010

**DOI:** 10.1371/journal.pone.0160766

**Published:** 2016-08-12

**Authors:** Lin Xiao, Dan-Li Zhang, Jill Torrie, Nathalie Auger, Nancy Gros-Louis McHugh, Zhong-Cheng Luo

**Affiliations:** 1 Public Health Department, Cree Board of Health and Social Services of James Bay, Mistissini, Quebec, G0W 1C0, Canada; 2 Department of Obstetrics and Gynecology, Sainte-Justine Hospital, University of Montreal, Montreal, Quebec, H3T 1C5, Canada; 3 Ministry of Education-Shanghai Key Laboratory of Children's Environmental Health, Xinhua Hospital, Shanghai Jiao-Tong University School of Medicine, Shanghai, 200092, China; 4 University of Montreal Hospital Research Centre, University of Montreal, Montreal, Quebec, H2X 0A9, Canada; 5 Research Division, First Nations of Quebec and Labrador Health and Social Service Commission, Wendake, Quebec, G0A 4V0, Canada; Public Health Agency of Canada, CANADA

## Abstract

**Background:**

Cree births in Quebec are characterized by the highest reported prevalence of macrosomia (~35%) in the world. It is unclear whether Cree births are at greater elevated risk of perinatal and infant mortality than other First Nations relative to non-Aboriginal births in Quebec, and if macrosomia may be related.

**Methods:**

This was a population-based retrospective birth cohort study using the linked birth-infant death database for singleton births to mothers from Cree (n = 5,340), other First Nations (n = 10,810) and non-Aboriginal (n = 229,960) communities in Quebec, 1996–2010. Community type was ascertained by residential postal code and municipality name. The primary outcomes were perinatal and infant mortality.

**Results:**

Macrosomia (birth weight for gestational age >90^th^ percentile) was substantially more frequent in Cree (38.0%) and other First Nations (21.9%) vs non-Aboriginal (9.4%) communities. Comparing Cree and other First Nations vs non-Aboriginal communities, perinatal mortality rates were 1.52 (95% confidence intervals 1.17, 1.98) and 1.34 (1.10, 1.64) times higher, and infant mortality rates 2.27 (1.71, 3.02) and 1.49 (1.16, 1.91) times higher, respectively. The risk elevations in perinatal and infant death in Cree communities attenuated after adjusting for maternal characteristics (age, education, marital status, parity), but became greater after further adjustment for birth weight (small, appropriate, or large for gestational age).

**Conclusions:**

Cree communities had greater risk elevations in perinatal and infant mortality than other First Nations relative to non-Aboriginal communities in Quebec. High prevalence of macrosomia did not explain the elevated risk of perinatal and infant mortality in Cree communities.

## Background

Perinatal and infant mortality rates are significantly elevated in Aboriginal populations compared to their non-Aboriginal counterparts even in developed countries including Australia, the U.S., and Canada [1–12]. In the Canadian province of Quebec, previous studies have found significantly elevated risks of perinatal and infant mortality for births to First Nations and Inuit mothers [3–5]. Births in Cree communities of Quebec are of particular interest in that they have the highest reported rate of infant macrosomia (about 35%, defined as birth weight >4000 g, or >90^th^ percentile for sex and gestational age) [13,14]. Maternal obesity, pre-gestational and gestational diabetes are major risk factors of infant macrosomia [15,16]. These risk factors are much more frequent in First Nations especially Cree than non-Aboriginal populations [17,18]. The high prevalence of maternal obesity and diabetes along with genetic factors could partly account for the high prevalence of infant macrosomia in Cree communities [14]. Macrosomic infants are more likely born to mothers with a relatively unhealthy intrauterine metabolic environment [15], and hence may be less healthy than normal birth weight infants. Therefore, it is plausible that macrosomia may partly account for the elevated risk of perinatal and infant mortality in Cree communities. It is unclear whether Cree births are at greater elevated risk of perinatal and infant mortality than other First Nations relative to non-Aboriginal births in Quebec, and what is the role of macrosomia. The present study sought to address this question in a large population-based birth cohort in Quebec, 1996–2010.

## Methods

### Study population

This was a population-based retrospective birth cohort study, based on the linked stillbirth, live birth and infant death database at the Institut de la Statistique du Québec (ISQ) for singleton births in Quebec, Canada 1996–2010, the most recent data available at the time of approval of the Aboriginal birth data linkage project by the Commission for Access to Information (CAI) [3]. The validity of Canadian vital records linkage has been well documented [19]. In Quebec, all vital events (live births, stillbirths weighing > = 500 g, and deaths) are required to be registered by law. The research birth cohort included all Aboriginal singleton births identified by mother tongue, residential postal code and municipality name, or Indian Registration System membership, and a random 20% sample of non-Aboriginal births in Quebec 1996–2010 [3]. The present study included singleton births at gestational age > = 20 weeks and birth weight > = 500 g in Cree (n = 5340), other First Nations (n = 10810) and non-Aboriginal (n = 229,960) communities. We excluded births in Inuit communities and Aboriginal births (identified by mother tongue or Indian Registration System membership) in non-Aboriginal communities from the analyses, because the present study was aimed to assess disparities in perinatal and infant mortality in Cree, other First Nations vs. non-Aboriginal communities. In adherence to the ISQ data confidentiality rules, birth numbers are reported to the nearest 10.

### Ethics statement

The study was approved by the research ethics board of Sainte-Justine hospital research center. Informed consented was not required because the study was based on administrative health databases. Patient records/information was anonymized and de-identified prior to data analysis.

### Identification of births from First Nations communities

Births to parents from First Nations communities were identified by residential postal code and municipality name. A birth was considered from a First Nation community if the mother’s residential postal code and municipality name corresponded to an Indian reserve in Quebec (about 90% residents are First Nations, according to the Canada 2006 census) on birth registration. There are a total of 41 First Nations communities (reserves) in Quebec, including 9 Cree communities (Waswanipi, Mistissini, Oujé-Bougoumou, Nemaska, Waskaganish Eastmain, Wemindji, Chisasibi, Chibougamau).

Because all Cree communities are in rural areas, we conducted sensitivity analysis to examine whether the risk disparities in perinatal and infant mortality (relative to non-Aboriginal communities) were similar if the comparisons were restricted to rural area, according to Statistics Canada’s recommended definition for rural area based on postal code and municipality code [20,21].

### Outcomes

The primary outcomes were perinatal mortality (stillbirths plus neonatal deaths) and infant mortality (neonatal deaths plus postneonatal deaths) rates [22]. We chose perinatal death rather than stillbirth or neonatal death as a primary outcome since it is sometimes difficult to determine whether a death at birth is a stillbirth or neonatal death for fetuses at the borderline of viability. The use of perinatal death could avoid potential misclassifications. Other outcomes included preterm birth (<37 completed weeks of gestation), small-for-gestational-age (SGA, birth weight <10^th^ percentile, based on the Canadian fetal growth standards[23]), large-for-gestational-age (LGA, birth weight >90^th^ percentile), low birth weight (<2500 g), high birth weight (>4000 g), stillbirth (fetal death 20 ≥weeks and ≥500 g), neonatal death (0–27 days of postnatal life) and postneonatal death (28–364 days of postnatal life).

Causes of infant death were categorized according to the classification of the International Collaborative Effort on Perinatal and Infant Mortality [24], based on International Classification of Diseases (ICD)-9 codes for deaths in 1996–1999 or ICD-10 codes for deaths in 2000–2010. The cause categories included congenital conditions, immaturity-related conditions, asphyxia, sudden infant death syndrome (SIDS), infection, external causes (e.g. injuries and accidents), other specific conditions, and remaining causes. Causes of stillbirth were not presented since there were no remarkable findings.

### Statistical analysis

Stillbirth and perinatal mortality rates were calculated per 1000 total births. Infant mortality rates were calculated per 1000 live births. Preterm, SGA and LGA birth rates were calculated per 100 total births (live births plus stillbirths). Crude risk ratios (RR) and risk differences (RD) with 95% confidence intervals (CI) were calculated to illustrate the magnitude of the disparities. Chi-square tests were used to examine the statistical significance of differences across study groups. Log-binomial regression models were fitted to obtain the crude and adjusted RR of perinatal and infant death comparing Cree and other First Nations to non-Aboriginal births accounting for repeated measures (multiple deliveries to the same mothers) in generalized estimating equations. The adjusted RRs were controlled for maternal characteristics including age (<20, 20–29, 30–34, ≥35 years), marital status (married, common-law union, single/divorced/widowed), parity (primiparous, multiparous), education [<11 y, 11 y (high school), 12–13 y (college), 14+ y (university)], and infant characteristics including sex, gestational age (week), and birth weight for gestational age (small <10^th^, appropriate 10-90^th^, large >90^th^ percentile, according to the Canadian sex- and gestational age-specific fetal growth standards [23]) (SGA, AGA, LGA). All data analyses were carried out using SAS, Version 9.2.

## Results

Maternal characteristics differed significantly between births in Cree, other First Nations and non-Aboriginal communities (**Table 1**). Mothers from Cree and other First Nations communities were much younger than mothers from non-Aboriginal communities. The proportion of mothers less than 20 years of age was over 4 times higher for births in Cree (19.3%) or other First Nations (20.2%) than non-Aboriginal (3.5%) communities. Mothers in Cree or other First Nations communities had much lower educational attainment. The proportion of mothers who had not completed high school was over 4 times higher for mothers in Cree (45.4%) or other First Nations (47.2%) than in non-Aboriginal (10.8%) communities, while the proportion of mothers with college or higher education was lower for mothers in Cree (11.5%) than in other First Nations (18.2%) or non-Aboriginal (57.9%) communities. The proportion of single, divorced or widowed mothers was substantially higher in Cree (21.6%) or other First Nations (34.1%) than in non-Aboriginal (9.1%) communities. Births to multiparous women were more common in Cree (70.1%) or other First Nations (66.1%) than non-Aboriginal (53.8%) communities. Birth weights were significantly higher for births in Cree (mean = 3772.5 g) and other First Nations (mean = 3549.5 g) vs. non-Aboriginal (mean = 3377.4 g) communities (p<0.0001).

**Table 1:** Characteristics of singleton births in Cree, other First Nations and Non-Aboriginal communities in Quebec, 1996–2010.

			Other		
		Cree	First Nations	Non-Aboriginal	P*
N, total births		5340	10810	229960	
Maternal age	Mean±SD	25.3±6.0	25.3±6.2	28.8±5.2	<0.001
years (%)	<20	19.3	20.2	3.5	<0.001
	20–34	72.2	71.0	82.4	
	>35	8.5	8.8	14.2	
Education	Mean±SD	10.5±2.5	10.7±3.0	13.9±3.1	<0.001
(%)	<11 y	45.4	47.2	10.8	<0.001
	11 y (high school)	26.3	22.9	15.3	
	12 -13y (some college)	16.9	11.7	16.0	
	≥14y (college or some university)	11.5	18.2	57.9	
Marital status	Married	39.0	17.8	40.7	<0.001
(%)	Common-law union	39.5	48.2	50.2	
	Single, divorced	21.6	34.1	9.1	
	or widowed				
Parity	Primiparous	30.0	33.9	46.2	<0.001
(%)	Multiparous	70.1	66.1	53.8	
Gestational age	Mean±SD	38.7±1.9	38.7±1.9	38.9±1.9	0.21
(week)					
Birth weight (g)	Mean±SD	3772.5±627.8	3549.5±604.8	3377.4±548.6	<0.001

*P values in Chi-square tests for differences in proportions or analysis of variance tests for differences in means among the three study groups.

The risks of preterm birth, infant macrosomia, perinatal and infant death were all significantly higher in Cree or other First Nations than non-Aboriginal communities (**Table 2**). Preterm birth rates were identical in Cree and other First Nations communities, and both were roughly 1.1 times as high as in non-Aboriginal communities. Compared to births in non-Aboriginal communities, HBW and LGA occurred 3.21 (95% CI 3.09, 3.34) to 4.06 (3.91, 4.21) times as frequently in Cree communities, and 1.94 (1.86, 2.01) to 2.34 (2.26, 2.44) times as frequently in other First Nations communities, while LBW (RR: 0.63 (0.54, 0.73) and 0.79 (0.72, 0.87)) and SGA (RR: 0.24 (0.20, 0.29) and 0.50 (0.46, 0.55)) birth rates were substantially lower in Cree and other First Nations vs. non-Aboriginal communities. Perinatal and infant mortality rates were 1.52 (1.17, 1.98) and 2.27 (1.71, 3.02) times as high for Cree (10.5 and 9.2 per 1000, respectively), and 1.34 (1.10, 1.64) and 1.49 (1.16, 1.91) times as high for other First Nations (9.3 and 6.1 per 1000, respectively) compared to non-Aboriginal (6.9 and 4.1 per 1000, respectively) births. Relative to non-Aboriginal births, the absolute risk differences (increases) in perinatal death and infant death were 3.5 (0.8, 6.3) per 1000 and 5.1 (2.5, 7.7) per 1000 for Cree births, and 2.3 (0.5, 4.1) per 1000 and 2.0 (0.5, 3.4) per 1000 for other First Nations births, respectively. Macrosomic birth, perinatal and infant mortality rates were the highest in Cree communities. Infant death occurred about 50% more frequently in Cree vs. other First Nations communities. Large risk elevations were observed in postneonatal mortality in both Cree (RR = 4.74 (3.17, 7.10)) and other First Nations (RR = 2.97 (2.07, 4.28)) communities. The relative disparities in perinatal and infant mortality rates comparing Cree or other First Nations to non-Aboriginal communities fluctuated and showed no apparent trends between 1996–2000 and 2006–2010 (data not shown). Analyses of cause-specific infant mortality rates revealed significant risk elevations for infant death due to SIDS, infections and external causes in Cree communities (4.07 (1.63, 10.16), 19.32 (9.51, 39.24) and 4.70 (1.43, 15.47) times, respectively), and due to SIDS and external causes in other First Nations communities (3.62 (1.79, 7.33) and 3.88 (1.50, 10.04) times, respectively).

**Table 2:** Birth outcomes, perinatal and infant mortality in Cree, other First Nations and non-Aboriginal communities, Quebec 1996–2010.

	Cree	Other FN	Non-Ab	RD (95% CI)	RR (95%CI)	P	RD (95% CI)	RR (95%CI)	P
	(A)	(B)	(C)	(A)—(C)	(A) vs. (C)		(B)—(C)	(B) vs. (C)	
N, total births	5340	10810	229960						
**Births, %**									
Preterm	7.1	7.1	6.4	0.7 (0.03, 1.4)	1.11 (1.01, 1.23)	0.035	0.7 (0.2, 1.2)	1.11 (1.04, 1.19)	0.003
SGA	2.1	4.3	8.6	-6.5 (-7.0, -6.2)	0.24 (0.20, 0.29)	<0.001	-4.3 (-4.7, -3.9)	0.50 (0.46, 0.55)	<0.001
LGA	38.0	21.9	9.4	28.1 (27.5, 30.1)	4.06 (3.91, 4.21)	<0.001	12.8 (12.0, 13.5)	2.34 (2.26, 2.44)	<0.001
Low birth weight	2.9	3.7	4.7	-1.7 (-2.2, -1.3)	0.63 (0.54, 0.73)	<0.001	-1.0 (-1.3, -0.6)	0.79 (0.72, 0.87)	<0.001
High birth weight	34.3	20.7	10.7	23.7 (22.3, 25.0)	3.21 (3.09, 3.34)	<0.001	10.1 (9.4, 10.9)	1.94 (1.86, 2.01)	<0.001
**Deaths, per 1000**									
Perinatal death	10.5	9.3	6.9	3.5 (0.8, 6.3)	1.52 (1.17, 1.98)	0.002	2.3 (0.5, 4.1)	1.34 (1.10, 1.64)	0.004
Stillbirth	6.2	6.3	3.9	2.3 (0.2, 4.4)	1.59 (1.13, 2.25)	0.008	2.4 (0.9, 3.9)	1.62 (1.27, 2.07)	<0.001
Infant death	9.2	6.1	4.1	5.1 (2.5, 7.7)	2.27 (1.71, 3.02)	<0.0001	2.0 (0.5, 3.4)	1.49 (1.16, 1.91)	0.002
Neonatal death	4.3	3.0	3.0	1.3 (-0.5, 3.0)	1.43 (0.94, 2.16)	0.090	-0.1 (-1.2, 1.0)	0.98 (0.69, 1.40)	0.923
Postneonatal death	4.9	3.1	1.0	3.9 (2.0, 5.8)	4.74 (3.17, 7.10)	<0.0001	2.1 (1.0, 3.1)	2.97(2.07, 4.28)	<0.001
**Cause-specific infant death, per 1000**									
Congenital anomalies	1.1	1.8	1.1	0.02 (-0.9, 0.9)	1.02 (0.45, 2.29)	0.960	0.7 (0.0, 1.5)	1.60 (1.00, 2.55)	0.046
Immaturity-related	0.4	0.6	0.8	-0.4 (-0.9, 0.2)	0.50 (0.13, 2.03)	0.327	-0.2 (-0.7, 0.3)	0.75 (0.33, 1.69)	0.484
Asphyxia	0.8	0.6	0.5	0.2 (-0.5, 1.0)	1.39 (0.52, 3.77)	0.511	0.01 (-0.4, 0.5)	1.03 (0.46, 2.35)	0.936
SIDS	0.9	0.8	0.2	0.7 (-0.1, 1.5)	4.07 (1.63, 10.16)	0.001	0.6 (0.1, 1.2)	3.62 (1.79, 7.33)	<0.001
Infections	2.1	0.3	0.1	2.0 (0.7, 3.2)	19.32 (9.51, 39.24)	<0.001	0.2 (-0.1, 0.5)	2.61 (0.79, 8.63)	0.104
External causes	0.6	0.5	0.1	0.4 (-0.2, 1.1)	4.70 (1.43, 15.47)	0.005	0.3 (-0.1, 0.8)	3.88 (1.50, 10.04)	0.003
Others	3.4	1.6	1.2	2.2 (0.6, 3.7)	2.79 (1.74, 4.49)	<0.001	0.4 (-0.4, 1.1)	1.30 (0.80, 2.13)	0.286

FN = First Nations; Non-Ab = Non-Aboriginal; SGA = small-for-gestational-age (birth weight <10^th^ percentile); LGA = large-for-gestational-age (birth weight >90^th^ percentile); RD = risk difference; RR = relative risk; CI = confidence interval.

Comparing macrosomic vs birth weight appropriate for gestational age (10^th^-90^th^ percentile) infants, there were no significant differences in perinatal mortality (6.4 vs. 8.8 per 1000, p = 0.44) and infant mortality (7.9 vs. 8.2 per 1000, p = 0.99) rates for births in Cree communities. In contrast, there were higher perinatal mortality rates (5.5 vs. 3.9 per 1000, p = 0.001), but no significant difference in infant mortality rates (2.6 vs. 2.1 per 1000, p = 0.13) comparing macrosomic vs weight appropriate for gestational age infants in non-Aboriginal communities.

The crude and adjusted RRs of perinatal and infant mortality comparing First Nations and other First Nations vs. non-Aboriginal populations from log-binomial regression models are shown in **Table 3**. Adjustment for maternal characteristic (age, education, marital status, parity), year of birth, infant sex and gestational age attenuated the risk disparities (as indicated by the RRs) in perinatal and infant death comparing Cree or other First Nations to non-Aboriginal communities. However, significantly elevated risks remained in infant death (adjusted RR = 1.76 (1.27, 2.43)) especially postneonatal death (adjusted RR = 2.89 (1.83, 4.55)) comparing Cree to non-Aboriginal communities, and in postneonatal death (adjusted RR = 1.73 (1.10, 2.71)) comparing other First Nations to non-Aboriginal communities. Further adjustment for year of birth, infant sex and gestational age accentuated the risk disparities (as indicated by the adjusted RRs) in perinatal and infant death comparing births in Cree or other First Nations to non-Aboriginal communities. Additional adjustment for birth weight for gestational age further widened the risk disparities. The risk elevations in perinatal and infant mortality were much greater for births in Cree communities than in other First Nations communities relative to non-Aboriginal communities.

**Table 3:** Crude and adjusted RRs of perinatal and infant mortality comparing births in Cree, other First Nations vs. Non-Aboriginal communities, Quebec 1996–2010.

			Model 1*		Model 2*		Model 3*	
	Crude		Adjusted		Adjusted		Adjusted	
	RR (95%CI)	P	RR (95%CI)	P	RR (95%CI)	P	RR (95%CI)	P
**Cree vs Non-Aboriginal**								
Perinatal death	1.52 (1.17, 1.99)	0.002	1.28 (0.93, 1.77)	0.131	1.72 (1.18, 2.51)	0.004	1.83 (1.25, 2.68)	0.002
Stillbirth	1.60 (1.13, 2.26)	0.009	1.38 (0.90, 2.12)	0.143	1.66 (1.05, 2.62)	0.023	1.84 (1.15, 2.95)	0.011
Infant death	2.28 (1.71, 3.04)	<0.001	1.76 (1.27, 2.43)	0.001	2.35(1.63, 3.38)	<0.001	2.49 (1.71, 3.61)	0.002
Neonatal death	1.43 (0.94, 2.17)	0.092	1.18 (0.73, 1.90)	0.507	1.59(0.90, 2.83)	0.112	1.61 (0.89, 2.89)	0.115
Postneonatal death	4.76 (3.18, 7.14)	<0.001	2.89 (1.83, 4.55)	<0.001	3.09(1.95, 4.88)	<0.001	3.45 (2.16, 5.52)	<0.001
**Other First Nations vs Non-Aboriginal**								
Perinatal death	1.34 (1.10, 1.65)	0.004	1.20 (0.93, 1.56)	0.160	1.36 (1.00, 1.87)	0.054	1.42 (1.03, 1.96)	0.031
Stillbirth	1.63 (1.27, 2.08)	<0.001	1.40 (1.00, 1.95)	0.049	1.53(1.07, 2.19)	0.020	1.63 (1.12, 2.63)	0.010
Infant death	1.49 (1.16, 1.92)	0.002	1.24 (0.92, 1.67)	0.162	1.34(0.95, 1.90)	0.095	1.41 (1.00, 2.00)	0.050
Neonatal Death	0.98 (0.69, 1.40)	0.923	0.99 (0.66, 1.49)	0.958	0.95(0.57, 1.58)	0.830	0.96 (0.57, 1.61)	0.869
Postneonatal death	2.98 (2.07, 4.29)	<0.001	1.73 (1.10, 2.71)	0.017	1.80(1.15, 2.83)	0.010	1.95 (1.24, 3.06)	0.004

RR = relative risk; CI = confidence interval; RRs were calculated from log-binomial regression models.

*Model 1, adjusted for maternal age, marital status, parity and education.

*Model 2 adjusted for all the variables in model 1, plus year of birth, infant sex and gestational age.

*Model 3, adjusted for all the variables in model 2, plus birth weight for gestational age.

The comparisons of birth outcomes and infant mortality in Cree, other First Nations and non-Aboriginal communities in rural areas **(Table 4)** showed similar patterns as those observed in the comparisons of the whole study birth cohort **(Table 2**). Significant risk elevations were observed in perinatal, infant and postneonatal mortality in both Cree (1.58 (1.20, 2.10), 2.47 (1.81, 3.37) and 5.61 (3.47, 9.07) times, respectively) and other First Nations (1.41 (1.13, 1.76), 1.62 (1.22, 2.15) and 3.58 (2.29, 5.59) times, respectively) relative to non-Aboriginal communities, and the risk elevations were greater in Cree than other First Nations relative to non-Aboriginal communities.

**Table 4:** Birth outcomes, perinatal and infant mortality in Cree, other First Nations and non-Aboriginal communities in rural areas, Quebec 1996–2010.

	Cree	Other FN	Non-Ab	RD(95%CI)	RR (95%CI)		RD(95%CI)	RR(95%CI)	
	(A)	(B)	(C)	(A)—(C)	(A) vs. (C)	P	(B)—(C)	(B) vs. (C)	P
N, total births	5340	10810	52790						
**Births, %**									
Preterm	7.1	7.1	6.6	0.6 (-0.2, 1.3)	1.09 (0.98, 1.20)	0.111	0.6 (0.03, 1.1)	1.09 (1.01, 1.17)	0.034
SGA	2.1	4.3	8.7	-6.7 (-7.1, -6.2)	0.24 (0.20, 0.29)	<0.001	-4.5 (-5.0, -4.1)	0.49 (0.45, 0.54)	<0.001
LGA	38.0	22.0	9.4	28.8 (27.5, 30.2)	4.06 (3.89, 4.24)	<0.001	12.9 (12.0, 13.7)	2.35 (2.25, 2.46)	<0.001
Low birth weight	2.9	3.7	4.9	-2.0 (-2.5, -1.5)	0.60 (0.51, 0.71)	<0.001	-1.2 (-1.6, -0.8)	0.76 (0.68, 0.84)	<0.001
High birth weight	34.3	20.8	10.6	23.9 (22.6, 25.2)	3.23 (3.09, 3.38)	<0.001	10.4 (9.5, 11.2)	1.96 (1.87, 2.05)	<0.001
**Deaths, per 1000**									
Perinatal death	10.5	9.3	6.6	3.8 (0.9, 6.6)	1.58 (1.20, 2.10)	0.001	2.6 (0.6, 4.6)	1.41 (1.13, 1.76)	0.003
Stillbirth	6.2	6.4	3.8	2.4 (0.2, 4.5)	1.64 (1.14, 2.37)	0.008	2.6 (1.0, 4.2)	1.70 (1.29, 2.24)	0.000
Infant death	9.2	6.1	3.8	5.5 (2.8, 8.1)	2.47 (1.81, 3.37)	<0.001	2.3 (0.7, 3.9)	1.62 (1.22, 2.15)	0.001
Neonatal death	4.3	2.9	2.9	1.4 (-0.4, 3.2)	1.51 (0.97, 2.34)	0.063	0.0 (-1.1, 1.1)	1.02 (0.70, 1.51)	0.904
Postneonatal death	4.9	3.1	0.9	4.1 (2.1, 6.0)	5.61 (3.47, 9.07)	<0.001	2.3 (1.2, 3.4)	3.58 (2.29, 5.59)	<0.001

FN = First Nations; Non-Ab = Non-Aboriginal; SGA = small-for-gestational-age (birth weight <10^th^ percentile); LGA = large-for-gestational-age (birth weight >90^th^ percentile); RR = relative risk; RD = risk difference; CI = confidence interval.

Table 5 presents perinatal and infant mortality rates comparing LGA or SGA to AGA infants within Cree, other First Nations and non-Aboriginal communities. LGA was not associated with higher risk of perinatal mortality in Cree communities, but was associated with higher risk of perinatal mortality in other First Nations and non-Aboriginal communities, and was not associated with infant mortality overall in Cree, other First Nations or non-Aboriginal communities. SGA was associated with higher risk of perinatal mortality in all the three groups, but the magnitudes of the risk increase were greater in Cree and other First Nations communities than in non-Aboriginal communities, and was associated with higher risk of infant mortality in Cree and non-Aboriginal communities.

**Table 5:** Perinatal and infant mortality rates comparing SGA or LGA to AGA births within Cree, other First Nations and non-Aboriginal communities in Quebec 1996–2010.

	Deaths,	per	1000						
	SGA	LGA	AGA	RD (95%CI)	RR (95%CI)		RD(95%CI)	RR (95%CI)	
	(A)	(B)	(C)	(A)—(C)	(A) vs. (C)	P	(B)—(C)	(B) vs. (C)	P
**Cree**									
Perinatal death	98.2	6.4	8.8	89.5 (34.3, 144.7)	11.22 (5.73, 21.96)	<0.001	-2.3 (-7.1, 2.4)	0.73 (0.38, 1.41)	0.352
Stillbirth	71.4	3.0	5.6	65.8 (18.0, 113.6)	12.69 (5.64, 28.57)	<0.001	-2.7 (-6.2, 0.9)	0.53 (0.21, 1.32)	0.165
Infant death	38.5	7.9	8.2	30.3 (-6.8, 67.4)	4.71 (1.67, 13.24)	0.001	-0.3 (-5.2, 4.7)	0.97 (0.52, 1.80)	0.922
Neonatal death	28.8	3.5	3.1	25.7 (-6.5, 57.9)	9.18 (2.56, 32.85)	<0.001	0.3 (-2.9, 3.5)	1.10 (0.42, 2.89)	0.842
Postneonatal death	9.9	4.5	5.0	4.9 (-14.6, 24.3)	1.96 (0.26, 14.65)	0.504	-0.6 (-4.4, 3.3)	0.89 (0.39, 2.00)	0.772
**Other FNs**									
Perinatal death	38.6	11.4	6.3	32.3 (14.8, 50.0)	6.14 (3.61, 10.44)	<0.001	5.1 (0.5, 9.8)	1.82 (1.42, 2.89)	0.011
Stillbirth	34.3	8.0	4.0	30.3 (13.7, 46.9)	8.53 (4.72, 15.44)	<0.001	4.0 (0.2, 7.9)	2.00 (1.13, 3.52)	0.015
Infant death	6.7	5.1	5.8	0.9 (-6.8, 8.6)	1.15 (0.36, 3.68)	0.816	-0.7 (-4.0, 2.7)	0.88 (0.47, 1.66)	0.695
Neonatal death	4.4	3.4	2.3	2.2 (-4.1, 8.4)	1.96 (0.46, 8.40)	0.359	1.1 (-1.4, 3.7)	1.50 (0.65, 3.45)	0.335
Postneonatal death	2.2	1.7	3.5	-1.3 (-5.9, 3.3)	0.63 (0.09, 4.60)	0.646	-1.8 (-4.0, 0.3)	0.48 (0.17, 1.38)	0.163
**Non-Aboriginal**									
Perinatal death	16.8	6.8	4.9	11.9 (10.0, 13.7)	3.41 (3.01, 3.86)	<0.001	1.9 (0.7, 3.0)	1.38 (1.16, 1.65)	<0.001
Stillbirth	11.2	4.0	2.9	8.3 (6.8, 9.8)	3.85 (3.3, 4.49)	<0.001	1.1 (0.2, 2.0)	1.37 (1.09, 1.73)	0.007
Infant death	8.0	3.6	2.9	5.1 (3.8, 6.3)	2.71 (2.28, 3.24)	<0.001	0.6 (-0.2, 1.5)	1.22 (0.96, 1.55)	0.110
Neonatal death	5.7	2.8	2.0	3.6 (2.6, 4.7)	2.8 (2.27, 3.46)	<0.001	0.8 (0.1, 1.5)	1.39 (1.06, 1.83)	0.017
Postneonatal death	2.4	0.8	1.0	1.4 (0.7, 2.1)	2.56 (1.85, 3.54)	<0.001	-0.2 (-0.6, 0.2)	0.83 (0.50, 1.39)	0.476

FN = First Nations; Non-Ab = Non-Aboriginal; SGA = small-for-gestational-age (birth weight <10^th^ percentile); LGA = large-for-gestational-age (birth weight >90^th^ percentile); RR = relative risk; RD = risk difference; CI = confidence interval.

## Discussion

### Main findings

We found that both Cree and other First Nations communities are at significantly elevated risks of perinatal and infant mortality relative to non-Aboriginal communities, but substantially greater risk elevations were observed in Cree than other First Nations communities. The high prevalence of macrosomia in Cree communities did not contribute to the high perinatal and infant mortality rates in Cree communities. The adjustment for birth weight small or large for gestational age did not diminish the risk disparities.

### Data interpretation and comparisons with previous studies

Elevated risks of perinatal and infant death have been reported in Aboriginal vs. non-Aboriginal populations even in developed countries,^1–12^ and in First Nations and Inuit populations in Quebec using mother tongue or place of residence to identify First Nations and Inuit births [3–5]. Cree births are uniquely characterized by very high prevalence of infant macrosomia [13,14]. The present study has clearly demonstrated greater risk elevations in perinatal and infant mortality in Cree than other First Nations relative to non-Aboriginal communities in Quebec, and that infant macrosomia is not a contributor to the elevated risk. LGA was a risk factor for perinatal death in other First Nations and non-Aboriginal infants, but not in Cree infants, and was not a risk factor for infant death in all the three groups.

The observed perinatal and infant mortality rates in Cree communities were not only substantially higher than the rates in non-Aboriginal communities, but also disappointingly higher than in other First Nations communities (especially for infant mortality, 1.5 times higher) in Quebec. This highlights the need for attention to improving perinatal and infant health in Cree communities. The largest mortality risk disparity was observed in postneonatal mortality comparing Cree to non-Aboriginal communities. Postneonatal mortality rate may reflect the impacts of socioeconomic conditions and infant care [25,26]. Aboriginal peoples had lower socioeconomic status, were much more likely to smoke tobacco and drink alcohol [27], and more frequently lack of convenient access to high-quality health care [28]. All Cree communities are located in the James Bay region, a remote area in northern Quebec, while about 75% of other First Nations communities are located in remote areas in northern Quebec. As a group, Cree communities are more remote than other First Nations communities. We speculate that socio-economic disadvantages, and more frequently lacked convenient access to high-quality health care and high prevalence of unhealthy lifestyle factors and maternal illnesses (e.g. diabetes) may partly explain the high perinatal and infant mortality rates in Cree communities.

An extremely large risk elevation was observed for infant death due to infections (19.32 times), and large risk elevations were observed for infant death due to SIDS (4.07 times) and injuries/external causes (4.70 times) in Cree relative to non-Aboriginal communities in Quebec. These risk elevations were much greater in Cree than other First Nations relative to non-Aboriginal communities, and deserve further studies. Such “preventable” infant deaths may be reduced through improvements in infant immunization coverage, promotion of safe infant sleep environment (back-to-sleep/supine sleep position, avoiding infant-adult bed-sharing) [29,30], and safe infant care environment (e.g. avoidance of smoking, alcohol and substance abuse, improvements in ventilation/ housing conditions) [30–32]. There is a need for further studies to understand the high risk of infant death due to infections in Cree communities. Unexpectedly, there was an elevated risk of infant mortality due to congenital anomalies in other First Nations but not Cree relative to non-Aboriginal communities. We speculated that this risk difference in infant mortality due to congenital anomalies might be related to differential exposures to environmental teratogens, and/or differential rates of early pregnancy screening and detection of birth defects resulting in termination of pregnancies with malformations. There is a need for more studies to understand the underlying causes.

Our study confirmed the high prevalence of infant macrosomia in Cree communities [13,14]. In contrast, SGA and LBW rates were much lower in Cree or other First Nation relative to non-Aboriginal births in Quebec. Our analyses showed that the much higher birth weights and high prevalence of macrosomia could not explain the high perinatal and infant mortality rates in Cree communities; the adjustment for birth weight (for gestational age) only widened the risk disparities. It is well-known that LBW and SGA infants are at high risk for perinatal and infant death. The high rates of perinatal and infant mortality despite low rates of SGA and LBW births in Cree and other First Nations communities suggest that poor access to high-quality care might partly account for their higher perinatal and infant mortality rates.

### Strengths and limitations

The main strength is the large population-based birth cohort. The main limitation is that some non-Aboriginal women living in First Nations communities would have been misclassified as Cree or other First Nations. However, such misclassifications should be small (less than 10% of residents are non-Aboriginal in First Nation reserves/communities in Quebec, according to the 2006 Census). Moreover, such misclassifications would only tend to bias the risk differences in perinatal and infant mortality rates in First Nations vs. non-Aboriginal communities towards the null. Another limitation is that the study could not address the causes of high birth weight such as maternal obesity and diabetes. However, these may be considered upstream risk factors that might affect perinatal and infant mortality risks through affecting fetal growth. Further studies are required to evaluate the contributions of these and other unmeasured risk factors to the elevated risks of perinatal and infant death in Aboriginal communities.

### Conclusions

Cree communities were at greater elevated risk of perinatal and infant mortality than other First Nations relative to non-Aboriginal communities in Quebec. The very high prevalence of macrosomia did not contribute to the substantially higher risk of perinatal and infant mortality in Cree relative to non-Aboriginal communities. There is strong need for interventions to improve perinatal and infant health in First Nations especially Cree communities in Quebec.

## Supporting Information

S1 STROBE Checklist Checklist of items that should be included in reports of cohort studies. (DOC)

S1 Clinical Studies Checklist PLOS ONE Checklist. (DOCX)
